# Classification of land use/land cover using artificial intelligence (ANN-RF)

**DOI:** 10.3389/frai.2022.964279

**Published:** 2023-01-06

**Authors:** Eman A. Alshari, Mohammed B. Abdulkareem, Bharti W. Gawali

**Affiliations:** ^1^Department of Computer Science and Information Technology, Thamar University, Dhamar, Yemen; ^2^Department of Computer Engineering Techniques, Al-Maarif University College, Ramadi, Iraq; ^3^Department of Computer Science and Information Technology, Dr. Babasaheb Ambedkar Marathwada University, Aurangabad, India

**Keywords:** artificial neural networks (ANN), random forest (RF), Sana'a city, neural-based, object-based

## Abstract

Because deep learning has various downsides, such as complexity, expense, and the need to wait longer for results, this creates a significant incentive and impetus to invent and adopt the notion of developing machine learning because it is simple. This study intended to increase the accuracy of machine-learning approaches for land use/land cover classification using Sentinel-2A, and Landsat-8 satellites. This study aimed to implement a proposed method, neural-based with object-based, to produce a model addressed by artificial neural networks (limited parameters) with random forest (hyperparameter) called ANN_RF. This study used multispectral satellite images (Sentinel-2A and Landsat-8) and a normalized digital elevation model as input datasets for the Sana'a city map of 2016. The results showed that the accuracy of the proposed model (ANN_RF) is better than the ANN classifier with the Sentinel-2A and Landsat-8 satellites individually, which may contribute to the development of machine learning through newer researchers and specialists; it also conventionally developed traditional artificial neural networks with seven to ten layers but with access to 1,000's and millions of simulated neurons without resorting to deep learning techniques (ANN_RF).

## 1. Introduction

Artificial intelligence (AI) has piqued the interest of many academics, researchers, and people working in various industries the efforts and inventions will propel the techniques of AI to success (Alshari and Gawali, 2022a). Machine-learning approaches include neural networks as a subset. They are essentially artificial intelligence systems that mimic connected “neuron units,” loosely modeled depending on the way neurons in the brain interact (Yuan et al., 2009). Computational models based on neural connections have been studied since the 1940's.

As computer processing power has increased, large sets of training data have been used to analyze computational models of input data based on neural connections. Since neural networks consist of many (“deep”) layers of simulated connected neural-based neurons, AI practitioners refer to these techniques as “deep learning.” Before the emergence of deep learning (machine-learning approaches), neural networks typically consisted of three to five layers and dozens of neurons (Girma et al., 2022). There are seven to 10 layers in a deep learning network, each housing thousands of artificial neurons (Alqadhi et al., 2021), and the neural networks work on a feedforward basis. This form of an artificial neural network is the most frequent (Ramdani et al., 2021). This arrangement only passes through the “hidden” levels from the input to the output. There are no loops in the network. In 1958, AI pioneer Frank Rosenblatt proposed the first single-neuron network (Kuemmerle et al., 2013). While the concept is not new, advances in processing power, training methodologies, and data availability have enabled better performance (Alshari and Gawali, 2021a).

A random forest (RF) classifier is a collection or group of classification and regression trees created through random resampling (Alqadhi et al., 2021) of the preparation set on datasets of comparable size to the preparation set, known as bootstraps (Rindfuss et al., 2004; Verburg et al., 2011). When a tree is built as the test set without a specific record from the initial dataset, several bootstraps are used (Shi et al., 2020). The speed of all test sets is developed to measure the speculation error (Alshari and Gawali, 2021b). It is resistant to overfitting and is expected to be more robust in the presence of anomalies and extremely high-dimensional boundary spaces than other AI algorithms (Singh et al., 2021). The SAGA GIS program was used in this study. It is freely available, open-source software. It has an intuitive user interface with various display options and a growing array of geoscientific approaches (Paul et al., 2021).

For the proposed approach (ANN_RF), there are several samples for only six main parameters of class LULC classification for creating model classes. The six parameters are as follows: high land, mountains, land area, built-up (vegetation and agriculture area), and bare land. The ANN classifier parameter for sample classes is limited in terms of parameters for sample classes since only samples are trained. The RF classifier's parameter for sample classes is a hyperparameter since classifying depends on several trees. Every tree contains several sub-trees, and every sub-tree has several sub-trees; hence, RF builds giant trees for identifying classes LULC in the region.

This study developed traditional artificial neural networks with seven to 10 layers using the proposed approach, ANN_RF, with access to thousands and millions of simulated neurons without resorting to deep learning techniques (Khwarahm, 2021). In this study, the neurons consisted of five inputs and three hidden layers. The output was 15 multi-classes from ANN, which was input for implementing the RF classifier that applied big number classes for each parameter of the LULC parameters. Because deep learning has various downsides, such as complexity, expense, and the need to wait longer for results, this creates a significant incentive and impetus to invent and adopt this notion to develop machine learning because it is simple. This study intended to increase the accuracy of machine-learning approaches for land use/land cover classification using Sentinel-2A, Sentinel-2B, and Landsat-8 satellites.

## 2. Literature review

Several pieces of literature on classifying changes in land use using machine learning methods were reviewed. As demonstrated by earlier research, machine learning is employed (Makwinja et al., 2021) because it is simpler, more adaptable, faster, and less expensive than deep learning and all other artificial intelligence techniques (Alshari and Gawali, 2022b). Deep comprehension problems and algorithms may be complex (Sarif and Gupta, 2021). The combination of object-based and neural-based ANN and RF classifiers has never been addressed, according to the survey results from this study (Xie et al., 2021).

### 2.1 ANN Classifier

This study covered the literature review about ANN classifier from previous studies as follow: (Mishra et al., 2017; Kadavi and Lee, 2018; Dibs et al., 2020; Dixit and Agarwal, 2020; Ekumah et al., 2020; Hamad, 2020; Kaya and Görgün, 2020; MohanRajan et al., 2020; Navin and Agilandeeswari, 2020; Rojas et al., 2020; Saddique et al., 2020; Xu et al., 2020; Angessa et al., 2021; Bhattacharya et al., 2021; Dede et al., 2021; Ghayour et al., 2021; Sang et al., 2021; Xie et al., 2021; Yusof et al., 2021; Ambinakudige and Intsiful, 2022; Fantinel et al., 2022; Gogumalla et al., 2022; Rizvon and Jayakumar, 2022; Theres and Selvakumar, 2022).

The literature review proved that Artificial Neural Network (ANN) is a supervised classification from machine learning based on traditional neural networks that contain limited hidden layers. It has been developed in deep learning using unlimited hidden layers called deep neural networks.

A previous study discovered that employing ANN is limited because of its subpar performance and that the results of applying ANN for classification accuracy vary. It suggests that ANN performs poorly in comparison to other algorithms.

For instance:

In their research using SVM, SAM, and ANN in Yusof et al. (2021), indicated that ANN produced the worst results (Dixit and Agarwal, 2020).

SVM was superior to ANN when Kadavi and Lee (2018) applied it.

Mishra et al. (2017) compared MLC, RF, SVM, and ANN and found that RF and SVM produced the best results.

Implemented SVM and ANN (Ambinakudige and Intsiful, 2022), with SVM having better results than ANN.

### 2.2. RF Classifier

This study covered the literature review about RF classifier from previous studies as follow: (Na et al., 2010; Wang et al., 2011, 2019; Rodriguez-Galiano et al., 2012; Eisavi et al., 2015; He et al., 2015, 2022; Ming et al., 2016; Sonobe et al., 2017; Nguyen et al., 2018; Thanh Noi and Kappas, 2018; Xu et al., 2018; Zhang et al., 2018; Abdullah et al., 2019; Gašparović et al., 2019; Márquez et al., 2019; Ge et al., 2020; Ghayour et al., 2021; Loukika et al., 2021; Rejith et al., 2021; Tan et al., 2021; Tassi et al., 2021; Vignesh et al., 2021; Behera et al., 2022; Girma et al., 2022; Huang and Wang, 2022; Karijadi and Chou, 2022; Matosak et al., 2022; Mwabumba et al., 2022; Sudhakar and Reddy, 2022).

The literature review indicated that the Random Forest algorithm had been widely used in classifying land changes, especially recently. It was superior in most studies in classification accuracy. The classifier's efficiency is due to it being based on an object-based technique. In object-based techniques, due to RF's excellent performance, this study determined that its use is significant, even though its classification accuracy results vary. Nevertheless, all results showed that the RF algorithm is the best machine-learning LULC classifier among the many researched algorithms. In the future, more testing in various climatic conditions will be necessary. Additionally, the findings showed that RF produced trustworthy and extremely accurate land cover maps over large areas with diverse and complicated geomorphologies and little human contact. We classified land use and land cover by comparing RF, ANN, and other classifiers. They discovered that RF performed better than ANN classifiers. For instance, the RF results are better than those from SVM and MLC, according to there and others in 2022 (Tan et al., 2021).

According to research conducted by Tan et al. (2021), RF outperforms the decision tree classifier, SVM, and ANN (Ghayour et al., 2021).

In their study published in Wang et al. (2019), discovered that the classification accuracies of RF, SVM, and k-NN were 86.61, 79.96, and 77.23%, respectively (Xu et al., 2018).

In Loukika et al. (2021), discovered that RF is the best classifier among SVM, RF, and CART in terms of overall accuracy using Landsat-8 and Sentinal-2A (Ge et al., 2020).

### 2.3. Multiple (hybrid) classifier approaches

This study covered the literature review about the hybrid classifier approach from previous studies as follows: (Malinverni et al., 2011; Wang et al., 2011; Kumar et al., 2013; Márquez et al., 2019; Munthali et al., 2020).

Previous research proved the originality of the hybrid work described in this study, although the ANN_RF was not previously visible. This study examined several hybrid techniques for classifying land change between 1970 and 2022. However, articles that combined object-based and neural computing (RF) could not be found.

For instance, a paper by Karijadi and Chou (2022), presented a hybrid technique based on the Complete Ensemble Empirical Mode that combines random forest (RF) with Long Short-Term Memory (LSTM; Wang et al., 2011).

Furthermore, He et al. (2022), carried out research on a hybrid deep-learning-based recurrent model (DGRN) to map the water clarity of worldwide lakes using Landsat-8 Operational Land Imager (OLI) pictures (Malinverni et al., 2011).

Huang and Wang (2022) created hybrid landscapes (Munthali et al., 2020) with enough cooling intensity to reduce the UHI effect successfully.

Wang et al. (2011) completed their work (Márquez et al., 2019) of using a hybrid technique combining supervised classification and principal component analysis (PCA) to identify dandified land changes.

Malinverni et al. (Kumar et al., 2013) presented a hybrid classification method. Compared to traditional pixel-based approaches, the suggested hybrid methodology allows for extracting additional LULC classes while significantly increasing classification accuracy. However, it is challenging to combine many classifiers. The limited literature indicates that more research on hybrid tactics is needed. It has been noted that the efforts to use mixed techniques are insufficient (Lo and Choi, 2004; Schepaschenko et al., 2011; Mahiny and Clarke, 2012; Singh et al., 2014; Vigneshl and Thyagharajan, 2014; Vignesh et al., 2016, 2022; Sturari et al., 2017; Vignesh and Thyagharajan, 2017; Mishra et al., 2018; Thyagharajan and Vignesh, 2019; de Deus et al., 2021; Regasa et al., 2021; Wambugu et al., 2021; Behera et al., 2022)^1^,^2^.

After 2000, hybrid approaches gained popularity. It was frequently used for classifying land cover. Research into hybrid classifiers is still needed to maximize the categorization accuracy of Landsat-8 pictures. They are regarded as more involved methods of classifying land cover. Because each classification system has benefits and drawbacks, choosing the best one can be challenging. For instance, the performance of supervised algorithms like maximum likelihood will improve with sufficient training points and normally distributed image data. However, such methods cannot yield reliable results in complex environments, necessitating additional methods like hybrid approaches. Early hybrid techniques, which began in the 1980's, soon after the development of supervised and unsupervised classification, were built using Landsat-8 images. However, the hybrid system has become more adaptable and potent because of enhanced classifiers.

## 3. The proposed approach

First, although this study used a multi-layered ANN, the initial neural networks only included five input levels and three hidden layers, and they only produced results for a single class. There were six fundamental parameters, each of which had several types. There were 5,000 samples and 100 trees for constructing model classes for six primary parameters: high land, mountains, land area, built-up vegetation, and bare area land.

By training data on land categories, ANN will identify the classes of land. It will compete with deep learning approaches. Typical artificial neural networks with seven to 10 layers are traditionally constructed with access to millions of simulated neurons in this study. An input layer was included in the neural network. There were two layers: a concealed layer and an output layer. The input layer received the input signals and passed them on to the next layer, which then deliverd the final prediction to the output layer.

The task at hand, perceptron, was a multi-layered perception system. A perceptron is made up of numerous neurons, which are the fundamental building blocks of the brain. Each circle symbolizes a neuron in simple terms. Perceptrons are a dense layer of vertically arranged neurons. One can now see each neuron in the image from a detailed perspective. Weights (w1, w2, w3) and biases were assigned to each neuron, and computations were carried out as follows:

(F = w1^*^x1 + w2^*^x2 + w3^*^x3), combination = bias + weights ^*^ input.

The input layer received the data first and then sent it to the hidden layers, where the interconnection between the two layers assigned weights to each input randomly. After bias was applied to each input neuron, the weighted total, a combination of weights and preferences, was conveyed through the activation function. The activation function determined which node should be used for feature extraction and then computes the output. The model weights were adjusted, and the prediction process was finished. The input node converted the data into numerical form. Each node was allocated a number, which denoted an activation value. The higher the number, the more intense the action.

The activation value was transferred to the next node based on weights and the activation function. Each node calculated and updated the weighted total (activation function) depending on the transfer function. It then performed a process called activation. This neuron was the only one that can perform this function. These nodes then chose whether or not to transmit the signal. The ANN adjusted the weights, which determined the signal extension. The activation traveled across the network until it reached the destination node.

Afterward, the RF classifier received the output ANN. The steps in the random forest approach were as follows: A random forest selected n random records from a dataset of output ANN records at random. Individual decision trees were created for each sample. Each decision tree generated a result. The simplest random forest with random features was made by randomly splitting a limited set of input variables at each node. Combining the work ANN classifier with the work RF classifier, the study concluded that ANN_RF and worked hyper-parameters yield the best split.

**Figure 1:**
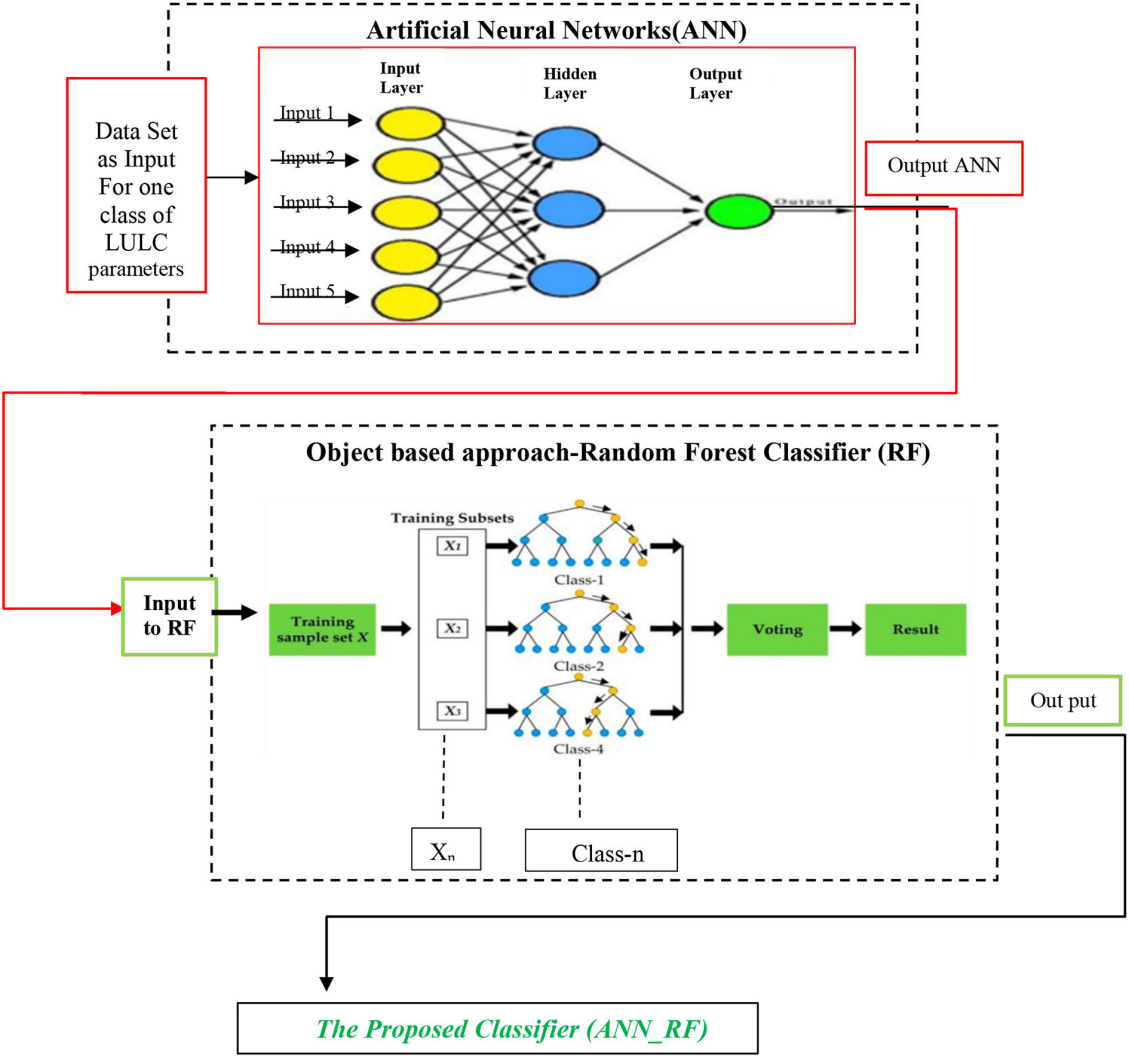
A workflow diagram for proposed work hybrid artificial neural networks (ANN_RF).

Finally, the suggested method sought to merge RF hyperparameters with ANN hyperparameters. Figure 1 shows hybrid artificial neural networks (ANN_RF).

Because of the merging of the two neural-based and object-based approaches, the number of layers in ANN_RF accessed thousands and millions of simulated neurons. The input to the RF classifier was the implementation of this novel method in software SAGA *via* the outputs of the artificial neural network. Then, rather than a single tree, each tree was built individually using the features of packing and randomness to produce a forest unrelated to what the trees expected.

## 4. Implementation of hybrid artificial neural networks (ANN_RF)

**Figure 2:**
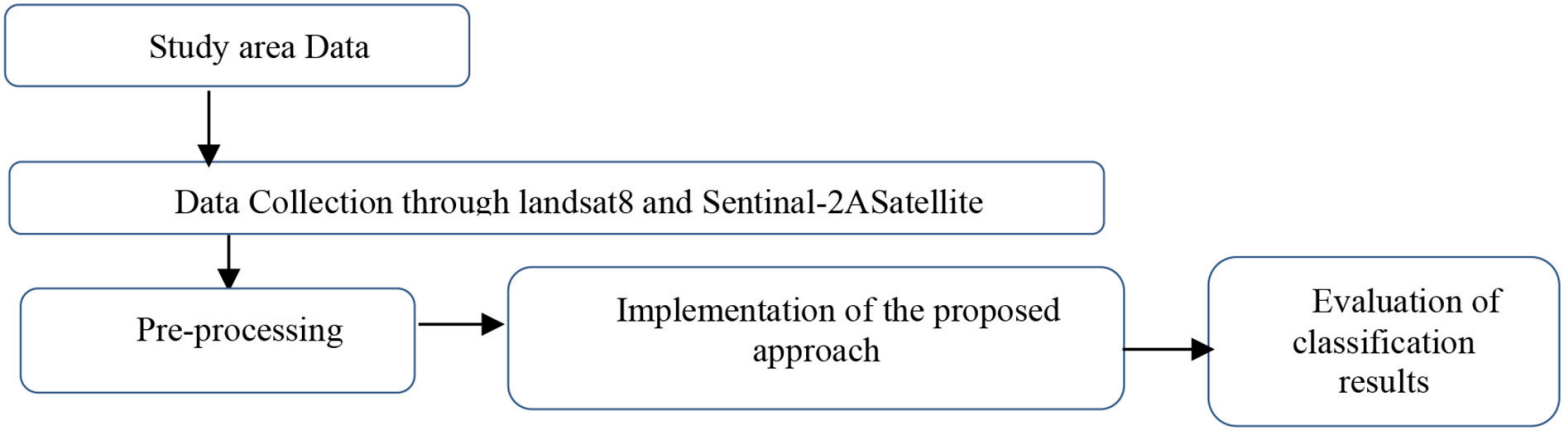
Workflow diagram for proposed methodology.

The process was conducted for LULC classification using the proposed classifier ANN_RF. It was provided at the outset of the study area data and was obtained afterward from multispectral satellites, Landsat-8 and Sentinel-2A satellites. This stage of preprocessing will be described in depth in the following section. LULC classification was done with SAGA software using the proposed classifier (ANN_RF) following its accuracy assessment. We used a change matrix with a polygon/grid in this study. We found LULC classification for Sana'a city with Landsat-8 resolution 30 m following in the down and sentimental-2A satellite. The fundamental parameter used in this study included six parameters: rocky area, mountains and high land, roads and land area, built-up area, vegetation, bare land, and agricultural area (see Figure 2).

### 4.1. Study area

**Figure 3:**
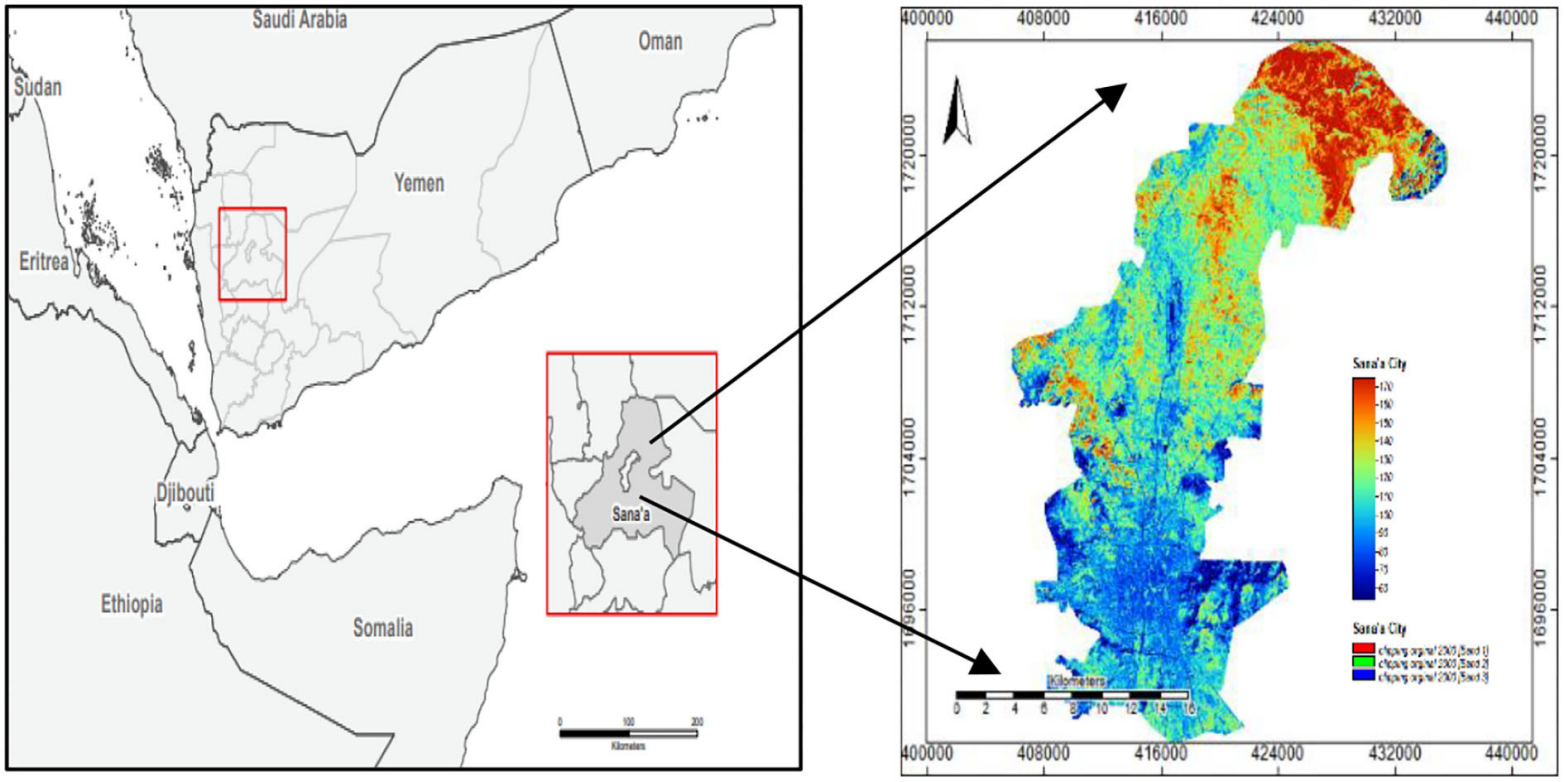
Location map of the case study.

Sana'a is Yemen's capital, the Sana'a governorate. It is situated at the height of 2,150 meters above sea level, on the equator's north line (15–21) and Greenwich's longitude (12–44) east (Bhattacharya et al., 2021). It is surrounded by two mountains (Jabal Naqum on the east and Jabal Eiban on the west) and the province (Bhattacharya et al., 2021). The city offers a unique environment around 2,200 meters above sea level (Angessa et al., 2021). Sana'a is Yemen's largest city and the administrative capital of the governorate. It is 2,300 m in height (7,500 ft). It is probably the most elevated capital close to the Sarawat Mountains. With an entire area of 21,084.06 km^2^ (49 sq mi), it has a population of around 3,937,500 (2012) (Alshari and Gawali, 2022b), as shown in Figure 3 of the cleared location case study.

### 4.2. Data collection

Images from a scientific agency in the Sana'a region were utilized in this investigation. The base map (Navin and Agilandeeswari, 2020) was created from survey pictures of the toposheet at a scale of 1:50,000. The data used in the current study were collected from the Landsat-8 satellite, which was launched in 2016. The calibration and comparison procedure for modifying land was made possible due to the 2016 data collected in December. In this study, the Landsat-8 satellite dataset contained 12 images, as shown in Table 1. Photos from a scientific agency in the Sana'a region were used in this investigation.

**Table 1:** Data collection from multispectral satellites.

**Date acquired**	**Sentinel-2A satellite**	**Landsat-8 satellite**
Sensor	Sentinel-2A.	The sensor is Operational Land Imager (OLI) and Thermal Infrared Sensor (TIRS).
Spatial of resolution	10 m	30 m
The time of the season	December	December

### 4.3. Preprocessing for LULC classification

**Figure 4:**
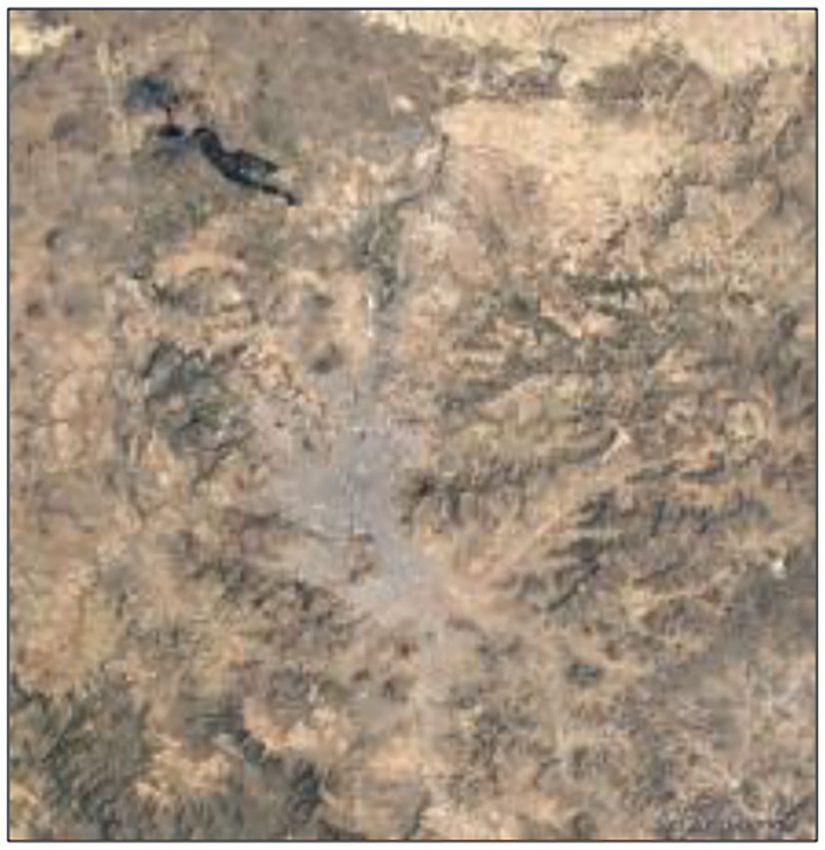
Sana'a area on the Google Maps.

The preprocessed data were separated into images in WGS84 or WGS84/UTM coordinate systems. Figure 4 identifies the information accurately after it was investigated using Google Maps and remote sensing technologies.

**Figure 5:**
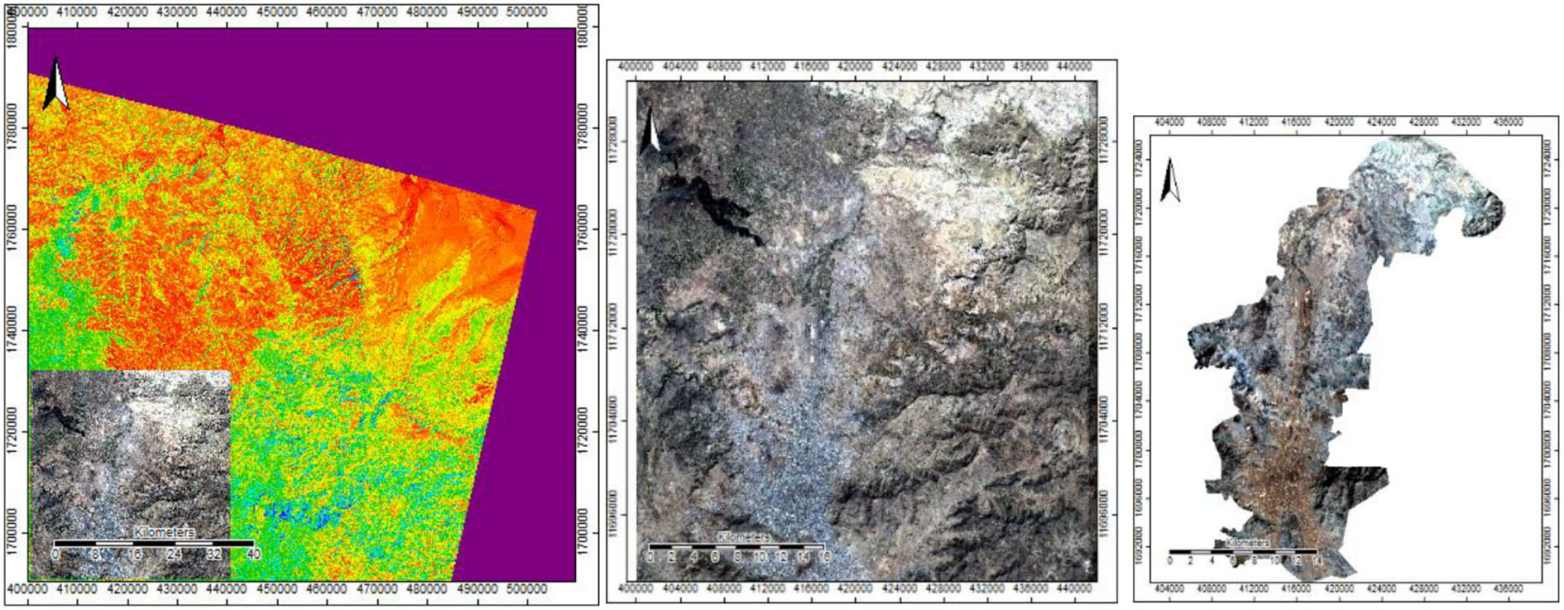
Dataset of Sentinel-2A satellite sensor with selection and clipping of area study in composite band 432.

**Figure 6:**
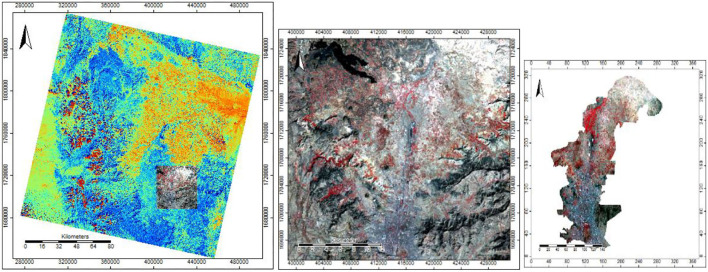
Dataset of Landsat-8 satellite sensor with selection and clipping of area study in composite band 432.

For the case study, multispectral pictures from Sentinel-2 and Landsat-8 were available (Figures 5, 6), demonstrating the preprocessing corrections for band 432. Valid data with geometric and radiometric corrections were included in the preprocessing. These processes improved satellite imagery for categorization and corrected degraded images to produce a more accurate scene representation.

### 4.4. LULC classification

**Figure 7:**
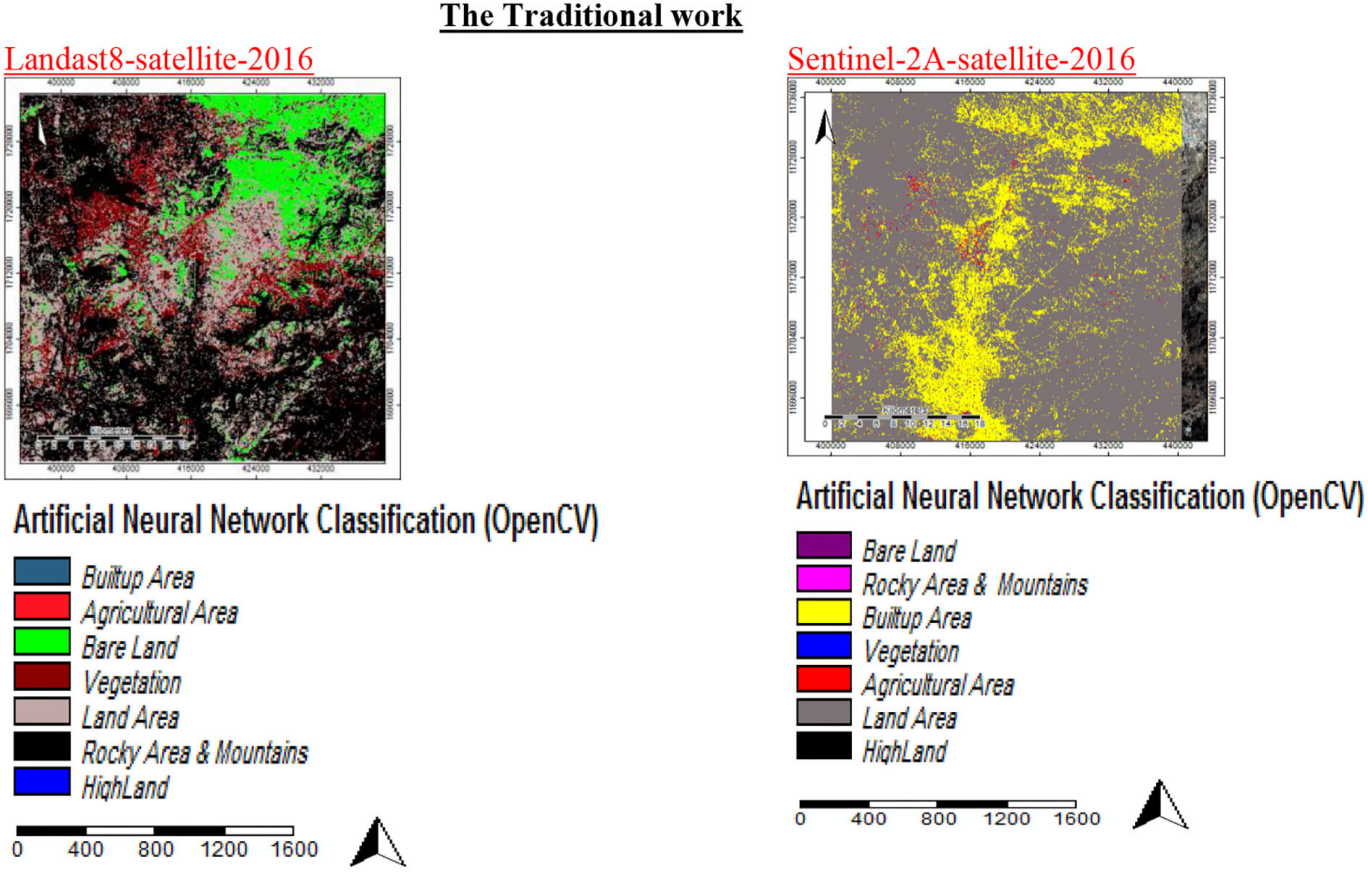
Classification of land use land cover with ANN classifiers alone.

**Figure 8:**
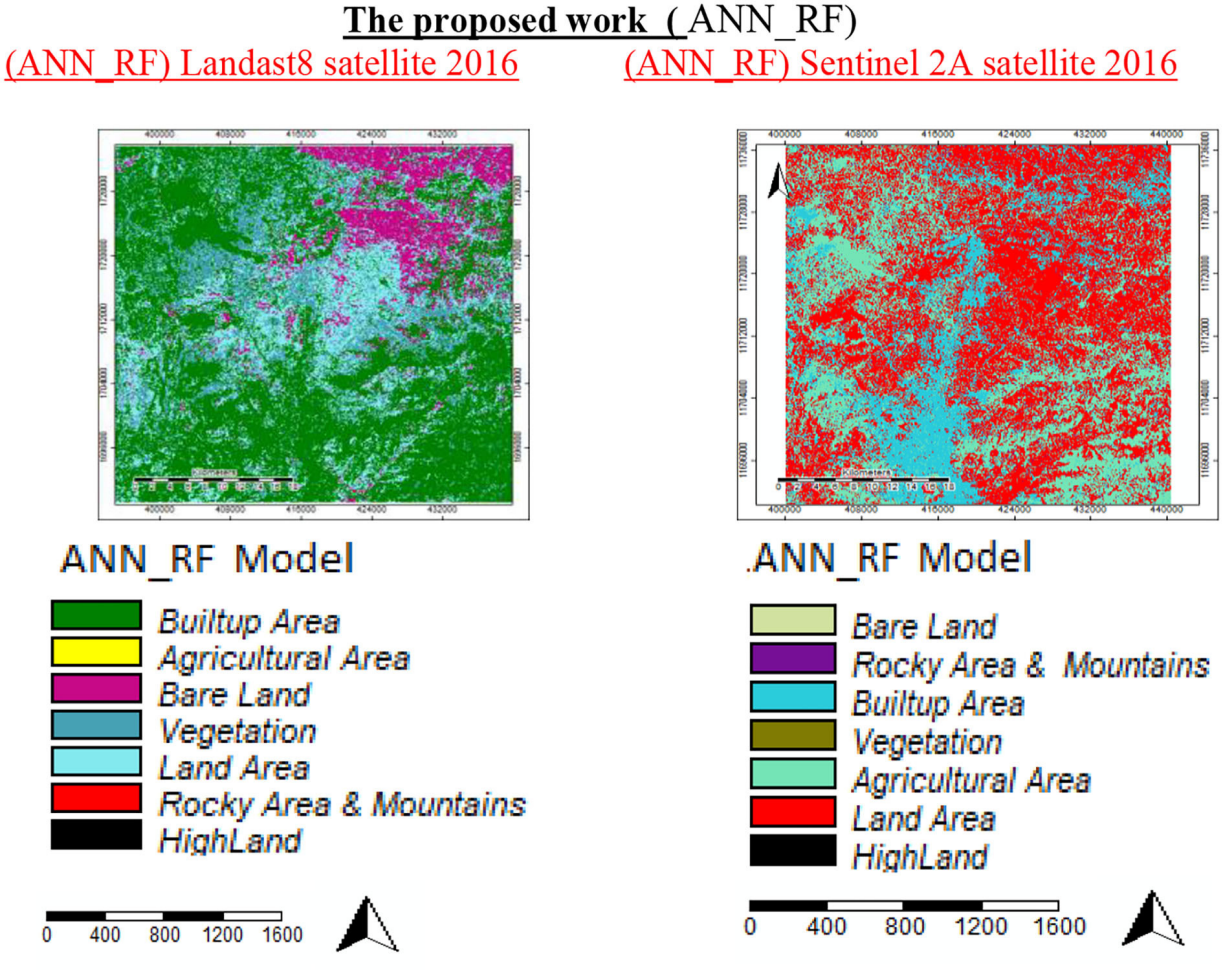
Classification of land use/land cover with the proposed work (ANN_RF).

This section explains the methodology applied in the general-level LULCC land use planning for Sana'a city and the specific findings derived using multispectral medium-resolution satellite data. Our analysis suggested that the LULC in Sana'a saw significant changes in 2016. This data source can be utilized to containerize the city sizes of Sana'a and, in the long term, contribute to territorial and global environmental models. In the database, there was a categorization model for LULC 2016. The RGB 432 band categorization was employed in this investigation—the ANN_RF. The neural networks used in this study had five input layers and three hidden layers. The output had six basic parameters and was only for one class. Each parameter had several different types. Table 2 shows 5,000 samples and 100 trees for six critical factors used to create model classes: high land, mountains, land area, built-up vegetation, and bare area land, with many subclasses, i.e., notes on SAGA software parameters. The categorization in these models was seven, but the processing and results in the parameter were six because merging area vegetation with farmed land created six classes. We created samples based on RGB color composites of Sentinel-2A photos, such as the vegetation class (red pixels in RGB = 432), which showed detailed changes in the region. For the proposed (ANN_RF) approach, numerous samples (hyper-parameter samples) of only six main parameters of the LULC classification were used to create model classes with detailed changes in the region. Figures 7, 8 are two different views of the same place.

**Table 2:** Description of LULC classes in the study area.

**LCLU classes**	**Description**
High land	High land may be remote settlements and clans with a long history and profound loyalties.
Mountains	A mountain is a raised section of the Earth's crust with steep sides and exposed bedrock.
Land area	The area in square kilometers of the land-based portions of conventional geographic regions is referred to as land area, which is the population of people. Not contains buildings, maybe streets, parks, roads or buildings crashed down, like this.
Builtup	Built-up maybe large buildings, small buildings, settlements, transportation land or places contain population people like banks, schools, hospitals, etc.
Vegetation	Space containing crops, fields, sparse grassland, a Temperate steppe, and a Temperate meadow.
Bare land	Bare soil, bare rocks, and land do not contain the population of people like the desert.

The types of hyperparameters used in this proposed model are max_sample and bootstrap. It improved the algorithms of machine learning (ANN and RF) with the help of the ANN_RF model, where types of hyperparameters of RF are as follows: max_depth in a random forest, the longest path between the root node and the leaf node was defined as the tree's maximum depth, min_sample_split, a random forest decision tree's minimum needed the number of observations in each node specified by this parameter; default = 2, max_leaf_nodes. These hyperparameters limited the tree's growth by requiring the splitting of nodes in the tree, min_samples_leaf. After splitting a node, this random forest hyperparameter set the minimum number of samples in the leaf node. Default = 1, estimators, the forest's total number of trees, max_sample; the max samples hyperparameters controlled how much of the original dataset was allocated to each tree, max_features; this was comparable to the maximum number of features offered to each tree in a random forest, the data point sampling method with or without replacement, and the criterion, the function for assessing the split's quality. The criteria “Gini” for Gini impurity and “entropy” for information gain were supported.

## 5. Evaluation of the ANN_RF model's performance

The proposed model's temporal complexity evaluated the ANN_RF model's performance. This study demonstrated that the proposed use of ANN_RF is a means to improve performance (both speed and accuracy). The time complexity of the (ANN_RF) model has three stages: First, the time complexity for ANN is calculated by the formula in Equation 1 (see text footnote ^1^). It shows the training time (in seconds) with ANN. As a result, the focus of ANN enhancement was on parameters and layers.

Mij*MjkMij*Mjk is simply O(i*j*k) O (i*j*k) (1) (time complexity for ANN) (see text footnote ^1^).

Then, it showed the time complexity in the case of RF. The time complexity for RF of constructing a complete unpruned decision tree was governed by Equation 2 (see text footnote ^2^), where n was the number of nodes in the tree: O (v ^*^ n log(n)) (2) (time complexity for RF) (see text footnote ^2^).

In the proposed technique, we merged the limited parameter (ANN) + hyperparameter (RF) = hyperparameter (ANN_RF) model in the proposed approach. It showed the time complexity of the proposed model, ANN_RF, with improved accuracy as 0.2 s, 0.3 s, 0.4 s, and 0.5 s. In the best case, the ANN_RF model had better time complexity than Equation 3, which was derived from Equations 1, 2, as shown below:

We explained the driven equation time complexity for (ANN_RF) model using a common extract factor between Equation 3 (time complexity for combination ANN ± RF).


(1) $$\begin{array}{l} \left[O\left(v*nlog\left(n\right)\right)\right)*\left(O\left(i*j*k\right)O\left(i*j*k\right)\right] \end{array}$$

(2) $$\begin{array}{l} \left[O\left(v*nlog\left(n\right)\right)\right)*\left(O\left(\left(i*j*k\right)\left(i*j*k\right)\right)\right] \end{array}$$

(3) $$\begin{array}{l} \left[O\left(v*nlog\left(n\right)\right)\right)*\left(O\left({\left(i*j*k\right)}^{2}\right)\right] \end{array}$$

(4) $$O[(v*nlog(n)*({\left(i*j*k\right)}^{2}]$$

(5) $$O((v*nlog(n)({\left(i*j*k\right)}^{2}).$$

On the other hand, a random forest randomly selected the rows and characteristics of the dataset using a bootstrapping technique. This randomization allowed a tree to have only a few samples and features while ensuring that all models and elements were considered in the other trees, restricting overfitting. The field determined the algorithm's output with the most votes in the event of a category variable and the average of all possible outcomes in the case of a numerical variable after generating several trees with different characteristics and samples.

It trained the model with many trees (for example, 1,000 trees) and then chose the best subset of trees to adjust the number of layers available to the ANN_RF trees. It was unnecessary to train a fresh random forest with different tree numbers each time. Slower learning is associated with higher accuracy. We reduced the number of estimators and increased the number of trees in the model to speed up the random forests.

## 6. Results

Tables 3–5 showed that the (ANN_RF) approach outperformed an ANN classifier using two satellites, Sentinel-2A and Landsat-8, better than the ANN classifier. This study successfully implemented the novel technique (ANN_RF). The result showed that improved classification accuracy for land use and land cover is possible. This study also found the proposed work for building machine learning with the overlap of entering ANN output into object-based approaches through the RF classifier to be practical. It was judged to be a high-accuracy classifier that enhanced ANN when it was single. Tables 4, 5 showed that ANN gave zero value in some classes, while (ANN_RF) gave some value to them. Further, the percentage of identified types in (ANN_RF) was better than in ANN.

**Table 3:** Overall accuracy and Kappa coefficient for ANN and proposed work (ANN_RF).

	**Sentinel-2A-2016**			**Landsat-8-2016**		
**No**	**Classifier**	**Overall accuracy**	**Kappa co-efficient**	**Classifier**	**Overall accuracy**	**Kappa co-efficient**
1	ANN	61.69%	0.736954	ANN	62.07%	0.434411
2	ANN_RF	82.52%	0.588419	ANN_RF	80.00%	0.719060

**Table 4:** Area and percentages LULC for ANN and (ANN_RF) to Sentinel-2A satellite.

**No**	**Name**	**ANN**		**ANN_RF**	
		**Area km^2^**	**Percentage %**	**Area km^2^**	**Percentage %**
1	Highland	120,003	01.00%	1,123,110	03.00%
2	Mountains	8,915,261	31.43%	148,753,130	39.14%
3	Land area	5,729,899	30.48%	130,431,000	04.69%
4	Vegetation	1,200,010	02.00%	287,400,010	03.15%
5	Bare land	3,614,288	03.00%	376,240,400	10.00%
6	Agricultural area	5,374,410	2.86%	311,200,010	09.00%
7	Built-up area	232,110	29.23%	11,098,100	31.02%
	Total=	18,796,889	100.00%	187,968,880	100.00%

**Table 5:** Area and percentages LULC for ANN and (ANN_RF) to Landsat-8 satellite.

**No**	**Name**	**ANN**		**ANN_RF**	
		**Area km^2^**	**Percentage %**	**Area km^2^**	**Percentage %**
1	Highland	129,800	02.00%	11,230	03.00%
2	Mountains	166,805,000	40.65%	101,668,050	33.69%
3	Land area	558,233,100	06.00%	475,654,500	02.00%
4	Vegetation	217,905,300	10.86%	227,799,900	11.35%
5	Bare land	214,587,000	10.68%	1,003,420	10.85%
6	Agricultural area	21,189	02.00%	98,710	01.00%
7	Builtup area	62,217,018	27.81%	108,622,170	38.11%
	Total=	2,007,405,900	100.00%	210,840,680	100.00%

There are elements affected by the results of land change classification and three factors:

i) Type of resolution satellite.
ii) Type of artificial intelligent classifier.
iii) Region types from the land use and land cover of the land selection. The processing stage is different from one classifier to another.

Every classifier in AI for land change classification has a process for running the algorithm.

## 7. Accuracy assessment

The confusion matrix and the A kappa coefficient were used for accuracy evaluation. A confused matrix of perplexity (sometimes called an error matrix) indicated how well a classification model or a classifier performed on a set of test data with known appropriate values (Ge et al., 2020). A confusion matrix was a tool for comparing the differences between two raster datasets. An error matrix was the most common technique for expressing the precision of the characterization result, the correctness of consumers and producers, and the insights acquired through mistake lattices (Abdullah et al., 2019). In the confusion matrix's columns, the classes to which pixels in an array corresponded for validation (ground truth) were employed. This study used SAGA GIS software to create a confusion matrix. The confusion matrix's results were divided into four groups. Tables 6–9 show that each group encompasses five categories of 2016 results. In this study, the SAGA GIS software used for LULC classification automatically created a confusion matrix and kappa coefficient with Excel for statistical computing values and overall accuracy, and the LULC image's kappa coefficient is shown in Table 3.

**Table 6:** Confusion matrix for ANN method of Sentinel-2A satellite 2016.

**Class**	**Highland**	**Mountains**	**Land area**	**Vegetation**	**Bare land**	**Agricultural area**	**Builtup area**	**SumUser**	**AccUser**
Highland	0	0	0	0	0	0	0	0	
Mountains	0	786	4	0	27	114	19	950	82.73684
Land area	444	33	518	203	68	210	1	1,477	35.07109
Vegetation	0	0	0	0	0	0	0	0	
Bare land	3	62	35	3	1,593	20	11	1,727	92.24088
Agricultural	0	24	2	0	6	43	0	75	57.33333
Builtup area	0	0	0	0	0	0	0	0	
Unclassified	0	0	0	0	0	0	0	0	0
SumProd	447	905	559	206	1,694	387	31		
AccProd	0	86.85083	92.66547	0	94.03778	11.11111	0		

**Table 7:** Confusion matrix for (ANN_RF) proposed work method of Sentinel-2A_satellite 2016.

**Class**	**Highland**	**Mountains**	**Land area**	**Vegetation**	**Bare land**	**Agricultural area**	**Builtup area**	**SumUser**	**AccUser**
Highland	0	0	0	0	0	0	0	0	
Mountains	446	859	350	188	114	380	10	2,347	36.59992
Land area	1	0	179	11	7	0	0	198	90.40404
Vegetation	0	0	6	6	3	0	0	15	40
Bare land	0	46	24	1	1,570	7	21	1,669	94.0683
Agricultural	0	0	0	0	0	0	0	0	0
Builtup area	0	0	0	0	0	0	0	0	
Unclassified	0	0	0	0	0	0	0		
SumProd	547	907	659	306	2,694	487	41		
AccProd	0	94.91713	32.02147	2.912621	92.68005	0	0		

**Table 8:** Confusion matrix for ANN method of Landsat-8 satellite 2016.

**Class**	**Highland**	**Mountains**	**Land area**	**Vegetation**	**Bare land**	**Agricultural area**	**Builtup area**	**SumUser**	**AccUser**
Highland	0	0	0	0	0	0	0	0	0
Mountains	90	413	20	0	1	0	993	1,517	27.22479
Land area	0	16	443	0	5	10	8	482	91.90871
Vegetation	0	0	6	135	0	115	4	260	51.92308
Bare land	0	0	1	0	1,078	0	0	1,079	99.90732
Agricultural	0	0	0	0	0	0	0	0	0
Builtup area	0	0	0	0	0	0	0	0	55.45814
Unclassified	0	0	0	0	0	0	0	0	0
SumProd	90	429	470	135	1,084	125	1,005		
AccProd	0	96.2704	94.25532	100	99.44649	0	0		

**Table 9:** Confusion matrix for (ANN_RF) proposed work method of Landsat-8 satellite 2016.

**Class**	**Highland**	**Mountains**	**Land area**	**Vegetation**	**Bare land**	**Agricultural area**	**Builtup area**	**SumUser**	**AccUse**
HighLand	0	0	0	0	0	0	0	0	0
Mountains	0	0	0	0	0	0	0	0	65.45814
Land Area	0	16	443	0	5	10	8	482	91.90871
Vegetation	0	0	6	135	0	115	4	260	51.92308
Bare Land	0	0	1	0	1,078	0	0	1,079	99.90732
Agricultural	0	0	0	0	0	0	0	0	0
Builtup Area	90	413	20	0	1	0	993	1,517	65.45814
Unclassified	0	0	0	0	0	0	0	0	0
SumProd	90	429	470	135	1,084	125	1,005		
AccProd	0	0	94.25532	100	99.44649	0	98.80597		

## 8. Discussion

Feature extraction from multispectral satellites is varied, such as being cheap, being free, covering a large region, and being more readily available. Additionally, the spectral bands in each image captured by multispectral satellites, as well as the spectral bands deduced from them, enabled us to access “hidden” data about features or objects that are used for image preprocessing, such as land use treatment, land cover classification, and more; however, it could not be detected. The features obtained from this study from the supplied satellite images—spatial, spectral, and spatial-spectral features—were retrieved.

An efficient method for analyzing texture features is the local binary pattern (LBP) method. It combines the advantages of statistical and structural approaches for texture analysis. Further, it increases the accuracy of machine learning approaches for land use/land cover classification using Sentinel-2A, and Landsat-8 satellites because deep learning has various downsides, such as complexity, expense, and the need to wait longer for results; this creates a significant incentive and impetus to invent and adopt the notion of developing machine learning approaches because it is simple.

We compared the outcomes of our suggested model with various other combinations, such as RF+SVM, MLC+SVM, and ANN+SVM, to assess the performance of the proposed method. We used two distinct satellite photos in this study. For our experiments, we specifically used Sentinel-2A and Landsat-8 satellite pictures. The Gabor filter extracts the crucial texture features from the raw image during the feature extraction stage.

For the supervised classification algorithm (object based) to classify data into various land use and land cover classes with ease during the classification phase. It was discovered that our suggested method produces results with greater accuracy than other methods. Finally, a clever edge detection algorithm separated the classes of land use and land cover from the LULC.

We discovered that combinations of classifiers had been discovered. However, we could not locate an ANN-RF classifier combination in the land use and land cover classification field. This shows that the scientific gap that was studied in this study has not yet been filled (Vigneshl and Thyagharajan, 2014; Vignesh et al., 2016, 2022; Vignesh and Thyagharajan, 2017; Thyagharajan and Vignesh, 2019).

## 9. Conclusion

This study concluded that the suggested study successfully implemented the novel technique—ANN_RF. It showed a significant result in classification accuracy for land use and land cover with Sentinel-2A and Landsat-8 satellites with the proposed approach, ANN_RF. Despite the advent of advanced technologies that have appeared over time for increased classification accuracy outcomes, this study recommends that future users and researchers continue research into the development of machine learning approaches. Further, it is recommened that researchers continue experimenting with merging these RF and ANN algorithms with other satellites and other time and environmental conditions. Through the aforementioned clarification, it is evident that this study's findings differ significantly from those of the earlier studies described in the literary survey part; this shows that the scientific gap studied here has not yet been bridged.

## Data availability statement

The datasets presented in this study can be found in online repositories. The names of the repository/repositories and accession number(s) can be found in the article/supplementary material.

## Ethics statement

Written informed consent was obtained from the individual(s), and minor(s)' legal guardian/next of kin, for the publication of any potentially identifiable images or data included in this article.

## Author contributions

All authors listed have made a substantial, direct, and intellectual contribution to the work and approved it for publication.

## Footnotes

^1^ https://ai.stackexchange.com/questions/5728/what-is-the-time-complexity-for-training-a-neural-network-using-back-propagation

^2^ https://www.quora.com/What-is-the-time-complexity-of-a-Random-Forest-both-building-the-model-and-classification

## References

[B1] Abdullah A. Y. M. Masrur A. Adnan M. S. G. Baky M. Al A. Hassan Q. K.. (2019). Spatio-temporal patterns of land use/land cover change in the heterogeneous coastal region of Bangladesh between 1990 and 2017. Remote Sensing 11, 790. 10.3390/rs11070790

[B2] Alqadhi S. Mallick J. Balha A. Bindajam A. Singh C. K. Hoa P. V. (2021). Spatial and decadal prediction of land use/land cover using multi-layer perceptron-neural network (MLP-NN) algorithm for a semi-arid region of Asir, Saudi Arabia. Earth Sci. Informat. 14, 1547–1562. 10.1007/s12145-021-00633-2

[B3] Alshari E. A. Gawali B. W. (2021a). Evaluation of the potentials and challenges of land observation satellites. Glob. Transit. Proc. 2, 73–79. 10.1016/j.gltp.2021.01.010

[B4] Alshari E. A. Gawali B. W. (2021b). Development of classification system for LULC using remote sensing and GIS. Glob. Transit. Proc. 2, 8–17. 10.1016/j.gltp.2021.01.00235624371

[B5] Alshari E. A. Gawali B. W. (2022a). Modelling for land use changes of Sana'a City of Yemen using MOLUSCE. J. Sens. 2022, 7419031. 10.1155/2022/7419031

[B6] Alshari E. A. Gawali B. W. (2022b). Analysis of machine learning techniques for sentinel-2A satellite images. J. Electr. Comput. Eng. 2022:9092299. 10.1155/2022/9092299

[B7] Ambinakudige S. Intsiful A. (2022). Estimation of area and volume change in the glaciers of the Columbia Icefield, Canada using machine learning algorithms and Landsat images. Remote Sens. Appl. 2022, 100732. 10.1016/j.rsase.2022.100732

[B8] Angessa A. T. Lemma B. Yeshitela K. (2021). Land-use and land-cover dynamics and their drivers in the central highlands of Ethiopia with special reference to the Lake Wanchi watershed. GeoJournal 86, 1225–1243. 10.1007/s10708-019-10130-1

[B9] Behera M. D. Sharma N. Chowdhary V. M. Shrestha D. G. (2022). “Hybrid approach for land use and forest cover classification in Sikkim Himalaya,” in Geospatial Technologies for Land and Water Resources Management. (Cham: Springer), 17–35. 10.1007/978-3-030-90479-1_2

[B10] Bhattacharya R. K. Das Chatterjee N. Das K. (2021). Land use and land cover change and its resultant erosion susceptible level: an appraisal using RUSLE and Logistic Regression in a tropical plateau basin of West Bengal, India. Environ. Dev. Sustainabil. 23, 1411–1446. 10.1007/s10668-020-00628-x

[B11] de Deus R. F. Tenedório J. A. Rocha J. (2021). “Modelling land-use and land-cover changes: a hybrid approach to a coastal area,” in Methods and Applications of Geospatial Technology in Sustainable Urbanism (Hershey, PA: IGI Global), 57–102. 10.4018/978-1-7998-2249-3.ch003

[B12] Dede M. Asdak C. Setiawan I. (2021). Spatial dynamics model of land use and land cover changes: a comparison of CA, ANN, and, ANN-CA register. Jurnal Ilmiah Teknologi Sistem Informasi 8, 38–49. 10.26594/register.v8i1.2339

[B13] Dibs H. Hasab H. A. Al-Rifaie J. K. Al-Ansari N. (2020). An optimal approach for land-use/land-cover mapping by integrating and fusion of multispectral landsat OLI images is a case study in Baghdad, Iraq. Water Air Soil Pollut. 231, 1–15. 10.1007/s11270-020-04846-x

[B14] Dixit A. Agarwal S. (2020). Super-resolution mapping of hyperspectral data using Artificial Neural Network and wavelet. Remote Sens. Appl. 20, 100374. 10.1016/j.rsase.2020.100374

[B15] Eisavi V. Homayouni S. Yazdi A. M. Alimohammadi A. (2015). Land cover mapping based on random forest classification of multitemporal spectral and thermal images. Environ. Monitor. Assess. 187, 1–14. 10.1007/s10661-015-4489-3 25910718

[B16] Ekumah B. Armah F. A. Afrifa E. K. Aheto D. W. Odoi J. O. Afitiri A. R. (2020). Assessing land use and land cover change in coastal urban wetlands of international importance in Ghana using intensity analysis. Wetlands Ecol. Manag. 28, 271–284. 10.1007/s11273-020-09712-5

[B17] Fantinel R. A. Pereira R. S. Benedetti A. C. P. Eugenio F. C. Marchesan J. Schuh M. S. (2022). Artificial intelligence and orbital images application for analysis of spatial land use and coverage patterns. Floresta 52, 313–322. 10.5380/rf.v52i2.79344

[B18] Gašparović M. Zrinjski M. Gudelj M. (2019). Automatic cost-effective method for land cover classification (ALCC). Comput. Environ. Urban Syst. 76, 1–10. 10.1016/j.compenvurbsys.2019.03.001

[B19] Ge G. Shi Z. Zhu Y. Yang X. Hao Y. (2020). Land use/cover classification in an arid desert-oasis mosaic landscape of China using remote sensed imagery: performance assessment of four machine learning algorithms. Glob. Ecol. Conserv. 22, e00971. 10.1016/j.gecco.2020.e00971

[B20] Ghayour L. Neshat A. Paryani S. Shahabi H. Shirzadi A. Chen W.. (2021). Performance evaluation of sentinel-2 and landsat 8 OLI data for land cover/use classification using a comparison between machine learning algorithms. Remote Sensing 13, 1349. 10.3390/rs13071349

[B21] Girma R. Fürst C. Moges A. (2022). Land use land cover change modeling by integrating artificial-neural-network with cellular automata-markov chain model in Gidabo river basin, main Ethiopian Rift. Environ. Challenges 2021, 100419. 10.1016/j.envc.2021.100419

[B22] Gogumalla P. Rupavatharam S. Datta A. Khopade R. Choudhari P. Dhulipala R.. (2022). Detecting soil pH from open-source remote sensing data: a case study of Angul and Balangir Districts, Odisha State. J. Indian Soc. Remote Sens. 9, 1–16. 10.1007/s12524-022-01524-9

[B23] Hamad R. (2020). An assessment of artificial neural networks support vector machines and decision trees for land cover classification using sentinel-2A. Data Sci. 8, 459–464. 10.12691/aees-8-6-18

[B24] He J. Harris J. R. Sawada M. Behnia P. (2015). A comparison of classification algorithms using Landsat-7 and Landsat-8 data for mapping lithology in Canada's Arctic. Int. J. Remote Sens. 36, 2252–2276. 10.1080/01431161.2015.1035410

[B25] He Y. Lu Z. Wang W. Zhang D. Zhang Y. Qin B.. (2022). Water clarity mapping of global lakes using a novel hybrid deep-learning-based recurrent model with Landsat OLI images. Water Res. 215, 118241. 10.1016/j.watres.2022.118241 35259557

[B26] Huang J. Wang Y. (2022). Cooling intensity of hybrid landscapes in a metropolitan area: relative contribution and marginal effect. Sustain. Cit. Soc. 79, 103725. 10.1016/j.scs.2022.103725

[B27] Kadavi P. R. Lee C. W. (2018). Land cover classification analysis of volcanic island in Aleutian Arc using an artificial neural network (ANN) and a support vector machine (SVM) from Landsat imagery. Geosci. J. 22, 653–665. 10.1007/s12303-018-0023-2

[B28] Karijadi I. Chou S. Y. (2022). A hybrid RF-LSTM based on CEEMDAN improves building energy consumption prediction accuracy. Energy Build. 259, 111908. 10.1016/j.enbuild.2022.111908

[B29] Kaya I. A. Görgün E. K. (2020). Land use and land cover change monitoring in Bandirma (Turkey) using remote sensing and geographic information systems. Environ. Monitor. Assess. 192, 1–18. 10.1007/s10661-020-08411-1 32535792

[B30] Khwarahm N. R. (2021). Using multitemporal satellite data, spatial modeling of land use and land cover change in Sulaimani, Iraq. Environ. Monitor. Assess. 193, 1–18. 10.1007/s10661-021-08959-6 33638037

[B31] Kuemmerle T. Erb K. Meyfroidt P. Müller D. Verburg P. H. Estel S.. (2013). Challenges and opportunities in mapping land use intensity globally. Curr. Opin. Environ. Sustainabil. 5, 484–493. 10.1016/j.cosust.2013.06.002 24143157 PMC3798043

[B32] Kumar P. Singh B. K. Rani M. (2013). An efficient hybrid classification approach for land use/land cover analysis in a semi-desert area using ${\rm ETM}{+} $ and LISS-III sensor. IEEE Sens. J. 13, 2161–2165. 10.1109/JSEN.2013.2251462

[B33] Lo C. P. Choi J. (2004). A hybrid approach to urban land use/cover mapping using Landsat 7 Enhanced Thematic Mapper Plus (ETM+) images. Int. J. Remote Sens. 25, 2687–2700. 10.1080/01431160310001618428

[B34] Loukika K. N. Keesara V. R. Sridhar V. (2021). Analysis of land use and land cover using machine learning algorithms on google earth engine for Munneru River Basin, India. Sustainability 13, 13758. 10.3390/su132413758

[B35] Mahiny A. S. Clarke K. C. (2012). Guiding SLEUTH land-use/land-cover change modeling using multicriteria evaluation: towards dynamic sustainable land-use planning. Environ. Plan. B 39, 925–944. 10.1068/b37092

[B36] Makwinja R. Kaunda E. Mengistou S. Alamirew T. (2021). Impact of land use/land cover dynamics on ecosystem service value—a case from Lake Malombe, Southern Malawi. Environ. Monitor. Assess. 193, 1–23. 10.1007/s10661-021-09241-5 34259941

[B37] Malinverni E. S. Tassetti A. N. Mancini A. Zingaretti P. Frontoni E. Bernardini A. (2011). Hybrid object-based approach for land use/land cover mapping using high spatial resolution imagery. Int. J. Geogr. Inform. Sci. 25, 1025–1043. 10.1080/13658816.2011.566569

[B38] Márquez A. M. Guevara E. Rey D. (2019). Hybrid model for forecasting of changes in land use and land cover using satellite techniques. IEEE J. Select. Top. Appl. Earth Observ. Remote Sens. 12, 252–273. 10.1109/JSTARS.2018.288561233040184

[B39] Matosak B. M. Fonseca L. M. G. Taquary E. C. Maretto R. V. Bendini H. D. N. Adami M. (2022). Mapping deforestation in cerrado based on hybrid deep learning architecture and medium spatial resolution satellite time series. Remote Sens. 14, 209. 10.3390/rs14010209

[B40] Ming D. Zhou T. Wang M. Tan T. (2016). Land cover classification using random forest with genetic algorithm-based parameter optimization. J. Appl. Remote Sens. 10, 35021. 10.1117/1.JRS.10.035021

[B41] Mishra V. N. Prasad R. Kumar P. Gupta D. K. Srivastava P. K. (2017). Dual-polarimetric C-band SAR data for land use/land cover classification by incorporating textural Information. Environ. Earth Sci. 76, 1–16. 10.1007/s12665-016-6341-7

[B42] Mishra V. N. Rai P. K. Prasad R. Punia M. Nistor M. M. (2018). Prediction of spatio-temporal land use/land cover dynamics in rapidly developing Varanasi district of Uttar Pradesh, India, using geospatial approach: a comparison of hybrid models. Appl. Geomat. 10, 257–276. 10.1007/s12518-018-0223-5

[B43] MohanRajan S. N. Loganathan A. Manoharan P. (2020). Survey on Land Use/Land Cover (LU/LC) change analysis in remote sensing and GIS environment: techniques and challenges. Environ. Sci. Pollut. Res. 27, 29900–29926. 10.1007/s11356-020-09091-7 32504427

[B44] Munthali M. G. Mustak S. Adeola A. Botai J. Singh S. K. Davis N. (2020). Modelling land use and land cover dynamics of Dedza district of Malawi using hybrid Cellular Automata and Markov model. Remote Sens. Appl. 17, 100276. 10.1016/j.rsase.2019.100276

[B45] Mwabumba M. Yadav B. K. Rwiza M. J. Larbi I. Twisa S. (2022). Using hybrid cellular automata-Markov model, analysis of land use and land-cover pattern to monitor dynamics of Ngorongoro world heritage site (Tanzania). Curr. Res. Environ. Sustainabil. 4, 100126. 10.1016/j.crsust.2022.100126

[B46] Na X. Zhang S. Li X. Yu H. Liu C. (2010). Improved land cover mapping using random forests combined with landsat thematic mapper imagery and ancillary geographic data. Photogrammetr. Eng. Remote Sens. 76, 833–840. 10.14358/PERS.76.7.833

[B47] Navin M. S. Agilandeeswari L. (2020). Multispectral and hyperspectral images based land use/land cover change prediction analysis: an extensive review. Multimedia Tools Appl. 79, 29751–29774. 10.1007/s11042-020-09531-z

[B48] Nguyen H. T. T. Doan T. M. Radeloff V. (2018). Applying random forest classification to map land use/land cover using Landsat 8 OLI. Int. Archiv. Photogrammetr. Remote Sens. Spatial Informat. Sci. 42, W4. 10.5194/isprs-archives-XLII-3-W4-363-2018

[B49] Paul S. Saxena K. G. Nagendra H. Lele N. (2021). Tracing land use and land cover change in peri-urban Delhi, India, over 1973–2017 period. Environ. Monitor. Assess. 193, 1–12. 10.1007/s10661-020-08841-x 33423184

[B50] Ramdani F. Setiawan B. Rusydi A. Furqon M. (2021). An artificial neural network approach to predict the future land use land cover of Great Malang Region. Indonesia 2021, 247. 10.20944/preprints202103.0247.v1

[B51] Regasa M. S. Nones M. Adeba D. (2021). A review on land use and land cover change in Ethiopian Basins. Land 10, 585. 10.3390/land10060585

[B52] Rejith R. G. Sundararajan M. Gnanappazham L. Seenipandi K. Ramaswamy S. (2021). GIS-based machine learning algorithms for mapping beach placer deposits in the southwest coast of India using Landsat-8 OLI images. J. Appl. Remote Sens. 16, 12011. 10.1117/1.JRS.16.012011

[B53] Rindfuss R. R. Walsh S. J. Turner B. L. Fox J. Mishra V. (2004). Developing a science of land change: challenges and methodological issues. Proc. Natl. Acad. Sci. 101, 13976–13981. 10.1073/pnas.0401545101 15383671 PMC521107

[B54] Rizvon S. S. Jayakumar K. (2022). Strength prediction models for recycled aggregate concrete using Random Forests, ANN and LASSO. J. Build. Pathol. Rehabil. 7, 1–10. 10.1007/s41024-021-00145-y

[B55] Rodriguez-Galiano V. F. Ghimire B. Rogan J. Chica-Olmo M. Rigol-Sanchez J. P. (2012). An assessment of the effectiveness of a random forest classifier for land-cover classification. ISPRS J. Photogrammetr. Remote Sens. 67, 93–104. 10.1016/j.isprsjprs.2011.11.002

[B56] Rojas F. Rubio C. Rizzo M. Bernabeu M. Akil N. Martín F. (2020). Land use and land cover in irrigated drylands: a long-term analysis of changes in the Mendoza and Tunuyán River basins, Argentina (1986–2018). Appl. Spatial Anal. Pol. 13, 875–899. 10.1007/s12061-020-09335-6

[B57] Saddique N. Mahmood T. Bernhofer C. (2020). Quantifying the impacts of land use/land cover change on the water balance in the afforested River Basin, Pakistan. Environ. Earth Sci. 79, 1–13. 10.1007/s12665-020-09206-w

[B58] Sang X. Guo Q. Wu X. Xie T. He C. Zang J.. (2021). The effect of DEM on the land use/cover classification accuracy of landsat OLI images. J. Ind. Soc. Remote Sens. 5, 1–12. 10.1007/s12524-021-01318-5

[B59] Sarif M. O. Gupta R. D. (2021). Spatiotemporal mapping of land use/land cover dynamics using. Remote Sensing and GIS approach: a case study of Prayagraj City, India (1988–2018). Environ. Dev. Sustainabil. 21, 1–33. 10.1007/s10668-021-01475-0

[B60] Schepaschenko D. McCallum I. Shvidenko A. Fritz S. Kraxner F. Obersteiner M. (2011). A new hybrid land cover dataset for Russia: a methodology for integrating statistics, remote sensing and in situ information. J. Land Use Sci. 6, 245–259. 10.1080/1747423X.2010.511681

[B61] Shi W. Zhang M. Zhang R. Chen S. Zhan Z. (2020). Change detection based on artificial intelligence: state-of-the-art and challenges. Remote Sens. 12, 1688. 10.3390/rs12101688

[B62] Singh R. K. Sinha V. S. P. Joshi P. K. Kumar M. (2021). A multinomial logistic model-based land use and land cover classification for the South Asian Association for Regional Cooperation nations using Moderate Resolution Imaging Spectroradiometer product. Environ. Dev. Sustainabil. 23, 6106–6127. 10.1007/s10668-020-00864-1

[B63] Singh S. K. Srivastava P. K. Gupta M. Thakur J. K. Mukherjee S. (2014). Appraisal of land use/land cover of mangrove forest ecosystem using support vector machine. Environ. Earth Sci. 71, 2245–2255. 10.1007/s12665-013-2628-0

[B64] Sonobe R. Yamaya Y. Tani H. Wang X. Kobayashi N. Mochizuki K. I. (2017). Mapping crop cover using multi-temporal Landsat 8 OLI imagery. Int. J. Remote Sens. 38, 4348–4361. 10.1080/01431161.2017.1323286

[B65] Sturari M. Frontoni E. Pierdicca R. Mancini A. Malinverni E. S. Tassetti A. N.. (2017). Integrating elevation data and multispectral high-resolution images for an improved hybrid Land Use/Land Cover mapping. Eur. J. Remote Sens. 50, 1–17. 10.1080/22797254.2017.1274572

[B66] Sudhakar C. V. Reddy G. U. (2022). Land use Land cover change Assessment at Cement Industrial area using Landsat data-hybrid classification in part of YSR Kadapa District, Andhra Pradesh, India. Int. J. Intellig. Syst. Appl. Eng. 10, 75–86. 10.18201/ijisae.2022.270

[B67] Tan J. Zuo J. Xie X. Ding M. Xu Z. Zhou F. (2021). MLAs Land cover mapping performance across varying geomorphology with Landsat OLI-8 and minimum human intervention. Ecol. Informat. 61, 101227. 10.1016/j.ecoinf.2021.101227

[B68] Tassi A. Gigante D. Modica G. Di Martino L. Vizzari M. (2021). Pixel-vs. object-based landsat 8 data classification in google earth engine using random forest: the case study of maiella national park. Remote Sens. 13, 2299. 10.3390/rs13122299

[B69] Thanh Noi P. Kappas M. (2018). Comparison of random forest, k-nearest neighbor, and support vector machine classifiers for land cover classification using Sentinel-2 imagery. Sensors 18, 18. 10.3390/s18010018 29271909 PMC5796274

[B70] Theres B. L. Selvakumar R. (2022). Comparison of landuse/landcover classifier for monitoring urban dynamics using spatially enhanced landsat dataset. Environ. Earth Sci. 81, 1–8. 10.1007/s12665-022-10242-x

[B71] Thyagharajan K. K. Vignesh T. (2019). Soft computing techniques for land use and land cover monitoring with multispectral remote sensing images: a review. Archiv. Comput. Methods Eng. 26, 275–301. 10.1007/s11831-017-9239-y

[B72] Verburg P. H. Neumann K. Nol L. (2011). Challenges in using land use and land cover data for global change studies. Glob. Change Biol. 17, 974–989. 10.1111/j.1365-2486.2010.02307.x27447350

[B73] Vignesh T. Kanimozhi K. V. Sathish R. Kumar R. P. Jeyavathana R. B. Ezhumalai P. (2022). “Land use and land cover classification using recurrent neural networks with shared layered architecture,” in 2022 International Conference on Computer Communication and Informatics (ICCCI), 1–10. 10.1109/ICCCI54379.2022.974083927295638

[B74] Vignesh T. Thyagharajan K. K. (2017). “Water bodies identification from multispectral images using Gabor filter, FCM and canny edge detection methods,” in 2017 International Conference on Information Communication and Embedded Systems (ICICES) (Piscataway, NJ: IEEE), 1–5. 10.1109/ICICES.2017.8070767

[B75] Vignesh T. Thyagharajan K. K. Beaulah Jeyavathana R. Kanimozhi K. V. (2021). “Land use and land cover classification using recurrent neural networks with shared layered architecture,” in International Conference on Computer Communication and Informatics (ICCCI). (Piscataway, NJ: IEEE).

[B76] Vignesh T. Thyagharajan K. K. Murugan D. Sakthivel M. Pushparaj S. (2016). A novel multiple unsupervised algorithm for land use/land cover classification. Indian J. Sci. Technol. 9, 1–12. 10.17485/ijst/2016/v9i42/99682

[B77] Vigneshl T. Thyagharajan K. K. (2014). “Local binary pattern texture feature for satellite imagery classification,” in 2014 International Conference on Science Engineering and Management Research (ICSEMR) (Piscataway, NJ: IEEE Explore), 1–6. 10.1109/ICSEMR.2014.7043591

[B78] Wambugu N. Chen Y. Xiao Z. Wei M. Bello S. A. Junior J. M.. (2021). A hybrid deep convolutional neural network for accurate land cover classification. Int. J. Appl. Earth Observ. Geoinform. 103, 102515. 10.1016/j.jag.2021.102515

[B79] Wang X. Gao X. Zhang Y. Fei X. Chen Z. Wang J.. (2019). Land-cover classification of coastal wetlands using the RF algorithm for Worldview-2 and Landsat 8 images. Remote Sens. 11, 1927. 10.3390/rs11161927

[B80] Wang X. Li Z. Gao Z. (2011). “Monitoring sandified land changes using multi-temporal Landsat TM/ETM+ Data in Dengkou County of Inner Mongolia, China,” in 2011 4th International Congress on Image and Signal Processing (Vol. 3). (Piscataway, NJ: IEEE), 1646–1651. 10.1109/CISP.2011.6100405

[B81] Xie F. D. Wu X. Liu L. S. Zhang Y. L. Paudel B. (2021). Land use and land cover change within the Koshi River Basin of the central Himalayas since 1990. J. Mountain Sci. 18, 159–177. 10.1007/s11629-019-5944-3

[B82] Xu X. Shrestha S. Gilani H. Gumma M. K. Siddiqui B. N. Jain A. K. (2020). Dynamics and drivers of land use and land cover changes in Bangladesh. Region. Environ. Change 20, 1–11. 10.1007/s10113-020-01650-5

[B83] Xu Z. Chen J. Xia J. Du P. Zheng H. Gan L. (2018). Multisource earth observation data for land-cover classification using random forest. IEEE Geosci. Remote Sens. Lett. 15, 789–793. 10.1109/LGRS.2018.2806223

[B84] Yuan H. Van Der Wiele C. F. Khorram S. (2009). An automated artificial neural network system for land use/land cover classification from Landsat TM imagery. Remote Sens. 1, 243–265. 10.3390/rs1030243

[B85] Yusof N. Shafri H. Z. M. Shaharum N. S. N. (2021). The use of Landsat-8 and Sentinel-2 imageries in detecting and mapping rubber trees. J. Rubber Res. 24, 121–135. 10.1007/s42464-020-00078-0

[B86] Zhang C. Sargent I. Pan X. Li H. Gardiner A. Hare J.. (2018). An object-based convolutional neural network (OCNN) for urban land use classification. Remote Sens. Environ. 216, 57–70. 10.1016/j.rse.2018.06.034

